# Azacitidine regulates DNA methylation of GADD45γ in myelodysplastic syndromes

**DOI:** 10.1002/jcla.23597

**Published:** 2020-10-20

**Authors:** Yanli Yang, Jun Li, Yinghua Geng, Lin Liu, Dianming Li

**Affiliations:** ^1^ Department of Hematology The First Affiliated Hospital of Bengbu Medical College Bengbu City China; ^2^ Department of Respiratory and Critical Care Medicine The First Affiliated Hospital of Bengbu Medical College Bengbu China

**Keywords:** azacitidine, GADD45γ, MDS

## Abstract

**Background:**

Myelodysplastic syndrome (MDS) is a heterogeneous clonal disease originated from hematopoietic stem cells. Epigenetic studies had demonstrated that DNA methylation and histone acetylation were abnormal in MDS. Azacitidine is an effective drug in the treatment of demethylation.

**Methods:**

RT‐PCR was performed to determine GADD45γ in 15 MDS clinical samples. Myelodysplastic syndrome cell lines SKM‐1 and HS‐5 were transfected with GADD45γ eukaryotic expression vector and/or GADD45γ shRNA interference plasmid, and treated with azacitidine. Proliferation and apoptosis were examined by CCK‐8 and Western blot analysis to confirm the function role of GADD45γ and azacitidine. The methylation level of GADD45γ gene was detected by bisulfite conversion and PCR.

**Results:**

This study found that GADD45γ gene was down‐expressed in MDS patients' bone marrow and MDS cell lines, and the down‐regulation of GADD45γ in MDS could inhibit MDS cell apoptosis and promote proliferation. Azacitidine, a demethylation drug, could restore the expression of GADD45γ in MDS cells and inhibit the proliferation of MDS cells by inducing apoptosis, which was related to prognosis and transformation.

**Conclusion:**

This study indicated that GADD45γ was expected to become a new target of MDS‐targeted therapy. The findings of this study provided a new direction for the research and development of new MDS clinical drugs, and gave a new idea for the development of MDS demethylation drug to realize precise treatment.

## Graphical abstract

Azacitidine Induce MDS cell apoptosis.

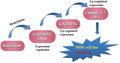

## 1 INTRODUCTION

The understanding of the biology and prognosis of myelodysplastic syndrome (MDS) have been made important progress. Myelodysplastic syndrome is a heterogeneous clonal stem cell disease of blood and bone marrow characterized by low hematopoietic function, which can lead to lethality or acute myeloid leukemia (AML). Its clinical manifestations were peripheral blood cell decrease, peripheral blood and bone marrow dysplasia/fibroblast, and clonal cytogenetic abnormality. The typical diagnosis was based on the presence of persistent cytopenia, dysplastic cells, and genetic markers. Common clinical problems included the diagnosis of MDS, the lack of new MDS drugs confirmation methods, and the lack of long‐term prospective randomized MDS clinical control to guide allogeneic blood and bone marrow transplantation.^1^ Recent studies had shown that there were epigenetic regulation (TET2, ASXL1, EZH2, DNMT3A, IDH1/2), RNA splicing (SF3B1, SRSF2, U2AF1, ZRSR2), DNA damage response (TP53), transcription regulation (RUNX1, BCOR, ETV6), and signal transduction (CBL, NRAS, JAK2) in MDS.^2^ The developments of small molecular drugs targeting specific molecular and the strategy to minimize their adverse reactions are the future development trend.^3^

DNA methylation, or the covalent addition of a methyl group to cytosine within the context of the CpG dinucleotide, had been widely found in mammalian genome. These effects included transcriptional repression and chromatin remodeling, X chromosome inactivation, and parasitic DNA suppression. Normal methylation patterns were frequently disrupted in tumor cells, and the promoter of a tumor suppressor gene was hypermethylation within gene silence and cell deletions or mutations.^4^ MDS‐specific therapies included drugs targeting abnormal DNA methylation and chromatin remodeling, regulating/activating the immune system to enhance tumor‐specific cellular immune response and reduce abnormal cytokine signaling, and blocking abnormal interactions between hematopoietic progenitor cells and stromal cells.^5^ For MDS treatment, research began to deepen to the gene level, such as a mutated cluster affecting three inositol‐specific genes, which was significantly related to the loss of response to azacitidine and linedoxamine treatment in high‐risk MDS patients.^6^ With the exploration of the pathogenesis of MDS, new potential therapeutic methods had been proposed, including hypomethylating agents with longer half‐life and exposure time, regulatory proteins such as antiapoptotic BCL2 protein, inhibition of PD‐1 or CTLA‐4, natural immunity, and targeted therapy with CD33/CD3 polyclonal antibody.^7^

Azacitidine (AZA) was the first drug approved by the US Food and Drug Administration (FDA) (May 2004) for the treatment of MDS, and had been granted the status of orphan drug. Azacitidine (Vidaza) had been approved by the European Union for use in patients with high‐risk MDS and AML.^8^ Azacitidine was a DNA methylation inhibitor, targeting at epigenetic gene silencing, which was used by cancer cells to inhibit gene expression against malignant phenotypes.^9^ At the same time, azacitidine was a valuable choice for the first‐line treatment of high‐risk MDS/AML patients.^10^ The pathogenesis of MDS was supposed to hypermethylation of specific DNA sequences. 5‐azacitidine and decitabine, which reactivate tumor suppressor gene transcription by DNA methylation, were the promising new agents.^11^ Abnormal DNA methylation was related to gene silencing. Hypomethylation drugs worked by inducing the re‐expression of epigenetic silencing genes.^12^ Hypermethylation of tumor suppressor gene had been considered as an important pathogenesis of MDS. Azacitidine, a pyrimidine nucleoside analogue, had the function of inhibiting DNA methyltransferase. It could be used as a treatment to prolong the survival of MDS patients, thus changing the natural history of these malignant tumors. The activity of azacitidine in MDS promotes its combination with other epigenetic modified to treat MDS and AML.^13^ In preclinical studies, azacitidine had low methylation/differentiation activity at low concentration, while high concentration was related to cytotoxic effect. In clinical trials, azacitidine not only improved MDS‐related cell reduction, but also delayed the transformation of leukemia, improved the quality of life, and improved the overall survival rate of many patients receiving AZA treatment.^14^ AZA could relieve the high‐risk MDS, which had become the frontline therapy for MDS that did not meet the conditions of allogeneic stem cell transplantation. AZA therapy, combination of AZA and other drugs, and prognosis therapy with AZA in specific cases could treat myeloproliferative neoplasms (MPN), chronic myelomonocytic leukemia (CMML), and AML.^15^ Epigenetic dysregulation was related to the pathogenesis of many malignant tumors, including MDS and AML. DNA methylation could lead to transcriptional silencing of tumor suppressor genes. By inhibiting DNA methyltransferase to re‐express these genes, it could treat benign and malignant diseases. In hematology, azacitidine and decitabine were widely used in clinic as demethylation drugs.^16^ 5‐azacitidine and decitabine had therapeutic effect on high‐risk MDS, and 5‐azacitidine could improve the survival rate of high‐risk MDS patients.^17^ The two structures were slightly different, and they were finally metabolized into 5‐aza‐CTP and 5‐aza‐dCTP through different metabolic pathways. Meanwhile, azacitidine was more effective in the treatment of MDS caused by mutation of DNA methylation pathway.^18^ AZA was the most effective drug in the treatment of MDS, but was only suitable for 50% of patients. The cause of resistance to AZA was debatable. Recent studies had found that AZA responders had more hematopoietic progenitor cells (HPCS) in cell cycle.^19^ Azacitidine changed the DNA methylation level of DLK1‐DIO3 region in the treatment of MDS and myelodysplastic‐related AML.^20^ Systematic evaluation of azacitidine in the treatment of MDS and AML had also been paid more and more attention.^21^ According to the latest research, the therapeutic effect of 5‐azacitidine in MDS was analyzed by detecting DNA methylation in peripheral blood.^22^ In the treatment of high‐risk MDS, the relative dose intensity of azacitidine had a significant impact on the survival rate of patients.^23^ After entering DNA, 5‐azacitidine based form covalent compounds with methyltransferase, and then, proteasome was recruited to degrade methyltransferase, so that highly methylated genes could be re‐expressed due to demethylation.^24^ Reactivate genes that were inactivated by DNA over methylation allowed cells to return to normal terminal differentiation, senescence, or apoptosis.^25^ At present, the prognosis evaluation system used in MDS is composed of morphology, clinical characteristics, and cytogenetics, but did not include molecular genetic data.

Based on the representative difference analysis of methylation sensitivity and 5‐aza‐2v‐deoxycytidine demethylation, CpG island of GADD45γ (Growth Arrest and DNA Damage 45γ) was the target sequence of hypermethylation gene. Like other GADD45 members, GADD45γ was widely expressed in all normal adult and fetal tissues. GADD45γ could inhibit cell growth and induce apoptosis in shock. The results showed that the ectopic expression of GADD45γ strongly inhibited the growth and colony formation of tumor cells in silenced cell lines.^26^ GADD45γ was an evolutionarily conserved protein that played important roles in growth suppression and apoptosis.^27^ GADD45γ played an important role in stress response, cell differentiation, and tumor inhibition by regulating cell proliferation and gene expression.^28^ GADD45 protein, including GADD45α, GADD45β, and GADD45γ, could participate in stress signal transduction. Through the interaction of other cell proteins (especially PCNA, p21, cdc2/cyclinB1, p38, and JNK stress response kinase) related to cell cycle regulation and cell response to stress, it led to cell cycle arrest, DNA repair, cell survival, aging, and apoptosis. GADD45 proteins could promote or inhibit tumor development and leukemia by using genetic engineering mouse model and bone marrow transplantation.^29^ In recent years, it had been found that myeloid differentiation (MyD) primary response and growth arrest DNA damage (GADD) genes consist of a group of overlapping genes, including GADD45, IRF‐1, EGR‐1, and MyD88.^30^ GADD45γ might be a functional tumor suppressor, which might play an important role in esophageal squamous cell carcinoma through the inactivation of proximal promoter methylation.^31^ GADD45γ, as a negative regulator of JAK STAT3 pathway, inhibited liver cancer by inducing cell aging. The decrease or absence of GADD45γ expression might be the reason why tumor cells or precancerous hepatocytes bypass cell aging.^32^ Recent studies had confirmed that GADD45γ can induce cardiomyocyte apoptosis in p38 MAPK‐dependent manner.^33^, ^34^ Meanwhile, the silencing or overexpression of GADD45γ was consistent with the effect of apoptosis and necrosis induced by GADD45γ.^35^

The molecular mechanism of 5‐aza‐2‐deoxycytidine (5‐aza‐2‐deoxycytidine) in multiple myeloma (MM) was to upregulate a large number of tumor‐related genes silenced by epigenetic genes.^36^ The multi‐targets and the lack of specificity made the treatment of MDS difficult.^25^ The frequency of GADD45γ methylation and the epigenetic change of GADD45γ might be related to the progress of diffuse large B‐cell lymphoma.^37^ MDS patients were rich in CpG island DNA hypermethylation, which led to the inactivation of tumor suppressor genes. Abnormal methylation of tumor suppressor genes was closely related to the occurrence and development of MDS. GADD45γ belonged to this kind of genes. There were many studies on the relationship between GADD45γ and tumor in solid tumors and AML; however, up to now, there is no report in MDS. MDS might also be due to hypermethylation of CpG island in the promoter region of GADD45γ gene, which led to the occurrence, development and transformation of MDS. With the development of whole‐genome sequencing technology, a large number of gene mutations were related to the occurrence, development, specific therapeutic response, and new targeted therapy of MDS, and the heterogeneity of MDS was being further clarified.

In this study, the expression of GADD45γ in MDS clinical samples was detected. We constructed GADD45γ eukaryotic expression vector and GADD45γ shRNA interference plasmid, and transfected MDS cell line SKM‐1 and American ATCC mouse normal bone marrow cell line (class B) HS‐5, respectively, to study the effect of GADD45γ on cell proliferation and apoptosis. Using tetracycline on gene expression system, the MDS cell line SKM‐1 was induced to overexpress GADD45γ by azacitidine. The effect of overexpression of GADD45γ on the proliferation of MDS cells in vitro was observed. In order to further understand the role of GADD45γ in MDS, to explore whether the gene can be a biomarker for predicting MDS demethylation drug treatment, and to provide new ideas for the research and development of MDS clinical new drugs and promoting the development of MDS precise treatment.

## 2 MATERIALS AND METHODS

### 2.1 Samples preparation

Sample collection: A total of 15 bone marrow samples from patients with MDS diagnosed for the first time at The First Affiliated Hospital of Bengbu Medical College from March 2018 to March 2019 were enrolled into this study. There are 8 men and 7 women with the mean age of 58.2 ± 9.7 years. Refer to the standard of diagnosis and curative effect of hematopathy for the diagnosis standard, and take 10 ml of bone marrow samples. This study was approved by the Human Ethics Committees Review Board at the The First Affiliated Hospital of Bengbu Medical College (S2018071).

Cell lines and reagents: MDS cell line SKM‐1 and human normal bone marrow cell line HS‐5 were purchased from ATCC (USA).

### 2.2 Cell line culture

The cells were cultured in RPMI 1640 medium containing 10% fetal bovine serum and placed in a cell incubator at 37°C and 5% CO_2_ saturated humidity until the logarithmic growth period.

### 2.3 Construction of p3XFLAG‐GADD45γ eukaryotic expression vector

Referring to the GADD45γ base sequence of GenBank mice, the GADD45γ primer (F forward: 5′‐TCTACGAGTCCGCCAAAGTC‐3′ and R forward: 5′‐GCACTTGCCACTGGTGTAGA‐3′) was designed by using primer premier 5.0. The total RNA of HS‐5 was extracted by Trizol method, took the oligo d(T) 18 as the reverse primer, took the extracted total RNA as the template, and reversed recorded the cDNA. Taking 1 μL DNA as template, the target gene was amplified by TaKaRa Ex TaqDNA polymerase. The PCR product was electrophoretic in 1.5% agarose gel, and the recovery procedure was purified according to TaKaRa gel recovery and Purification Kit. The fragments of GADD45γ coding region and the vector were digested with Kpn I and BamH I, respectively, and the fragments of gelled harvest vector and GADD45γ were digested with T4 DNA ligase. The products were transformed into DH5a‐sensitive cells, coated with plates (resistant to ampicillin), and grown at 37°C for 16‐24 hours, and about 500 spots were grown. Four spots were picked out and dissolved in 1ml LB medium with resistance to ampicillin for 3‐4 hours. one μl of bacterial solution was taken as culture medium Four clones amplified GADD45γ fragment for PCR. The positive bacterial solution was shaken in 4 mL ampicillin resistant LB liquid medium, 37°C overnight. According to the operating procedures of the kit, p3XFLAG‐GADD45γ was extracted and purified, and the plasmids were digested by enzyme, identified by electrophoresis, and then extracted and sequenced.

### 2.4 Construction of GADD45γ shRNA interference vector

The GADD45γ shRNA compatible with pLKO.1 plasmid was searched at http://www.sigmaaldrich.com/, and the GFP shRNA oligos included the sequence of sh‐GADD45γ in the experimental group and the sequence of shGFP in the control group. The sequence was synthesized by Shanghai biotechnology. The plasmid pLKO.1‐TRC was digested by EcoR I and Age I, the digested plasmid was recovered, and the double‐stranded interference sequence formed by annealing was linked by T4 DNA ligase and then transferred into human Stbl3 supersensitive cells for bacterial liquid PCR screening. The positive bacterial liquid was taken and then expanded for culture. Plasmid extraction was used to obtain a pure lentivirus plasmid.

### 2.5 Plasmid transfection into SKM‐1 cells

The MDS cell line SKM‐1 was inoculated into a 6‐well plate for cell culture. When the cell fusion rate reached 70%‐80% in 24 hours, the cells were transfected with p3XFLAG‐GADD45γ, p3XFLAG (as negative control), pLKO.1‐shRNA, and pLKO.1‐shGFP at a concentration of 2.5:1 by Lipofectamine^TM^ 2000. After incubation for 6 hours, the protein and RNA were extracted, respectively. Lipofectamine^TM^ 2000 was replaced by DMEM containing 10% fetal bovine serum for further culture.

### 2.6 Detection of GADD45γ mRNA expression by RT‐PCR

MDS cells were inoculated on the 6‐well plate. When the cell fusion rate reached 70%‐80%, p3XFLAG‐GADD45γ plasmid and p3XFLAG plasmid were transfected with Lipofectamine^TM^ 2000, and the cells were cultured for 72 hours. The third‐generation HS‐5 cells were inoculated on the 6‐well plate, and the cell fusion rate reached 60%‐70%. The qPCR primer sequences were listed in Table 1. When the lentivirus was infected, the culture solution of polyamine was changed, and 500 μL virus solution was added into each hole, the final concentration of polyamine was 8 μg/mL 48 hours later, and the minimum killing concentration of puromycin was used to screen the stable interfering cell line and continue to culture for 2 generations. The total RNA was extracted by Trizol method, and the cDNA was inversely obtained. RT‐PCR was used to calculate the mRNA expression of the target gene after standardization by the internal reference gene.

**TABLE 1:** The q‐PCR primer sequences

Gene	Forward primer	Reverse primer
GADD45γ for SKM‐1	5'‐AACTAGCTGCTGGTTGATCG‐3'	5'‐CGTTCAAGACTTTGGCTGAC‐3'
GADD45γ for HS‐5	5'‐CTCTGGAAGAAGTCCGTGGC‐3'	5'‐CAATGTCGTTCTCGCAGCA‐3'
GAPDH	5'‐GTCGGAGTCAACGGATTTGG‐3'	5'‐ATGGTGGTGAAGACGCCAGT‐3'

### 2.7 Detection of apoptosis‐related protein expression by Western blot

The MDS cell line SKM‐1 was inoculated on the 6‐well plate. When the cell confluence was 70%‐80%, the p3XFLAG‐GADD45γ plasmid and p3XFLAG plasmid were transfected with Lipofectamine^TM^ 2000. Seventy‐two hours later, the third‐generation FDC‐P1 was inoculated on the 6‐well plate, and the fusion degree of the cells was 60%‐70%. When the lentivirus was infected, the culture medium containing polyamine was changed, and 500 μL lentivirus solution was added into each hole, so that the final concentration of polyamine was 8 μg/mL, 48 hours, and the minimum killing concentration of puromycin was selected to stabilize the interfering cell lines. The 2nd generation was continuously cultured, and the protein lysate was dissolved in 30 minutes by ice bath and centrifugated, and then, the 50 g protein thereof was extracted by dodecyl sulfonate‐polyacrylamide gel electrophoresis. The wet spinning method was transferred to the polyvinylidene fluoride membrane. The 5% skimmed milk powder was sealed at room temperature, and the 1H was added at room temperature (Caspase‐3, Caspase‐7, and Caspase‐9). Caspase‐3, Caspase‐7, and Caspase‐9 were diluted with 1:1000 and incubated overnight at 4°C. After TBST washing, HRP‐labeled second antibody (diluted with 1:5000) was added. After incubation for 1H at room temperature, TBST membrane was rinsed for three times, the positive bands were displayed with chemiluminescent reagent, and the images were processed and analyzed with gray‐scale analysis software.

### 2.8 Cell proliferation testing

CCK‐8 method was used to detect the cell survival rate. SKM‐1 cell line transfected with 24‐hours MDS and FDC‐P1 cell infected with virus were inoculated into 96‐well plate (100 μL/well) with a density of approximate 5 × 10^3^ cells/mL. the control group was set up with 5 multiple wells in each group. After incubation for 48 hours, CCK‐8 of 10 μL was added to continue incubation for 2 hours. The absorbance (a) value of each hole was measured at 450 nm of enzyme‐linked immunosorbent assay. Calculate the survival rate of those cells.

### 2.9 Determination of the optimal concentration of GADD45γ expression induced by azacitidine

The experiment was divided into 6 groups. The final concentration of azacitidine in each experimental group was set as 0, 0.25, 0.5, 0.75, 1, and 5 μmol\L, respectively. After 24 hours, the expression of Turbo RFP was observed under the inverted fluorescence microscope (Zeiss). After determining the optimal concentration of azacitidine, Western blot was used to detect the expression of GADD45γ induced by azacitidine.

### 2.10 Detection of methylation level of GADD45γ gene

Genomic DNA isolated from SKM‐1 cells and three controls with AZA was used for analysis of the methylation. Referring to previous literature,^20^ bisulfite conversion was performed using EpiTect Bisulfite Kit. Amplicons were generated using FastStart High Fidelity PCR System and purified with Agencourt AMPure beads. The purified PCR products were sequenced using the Roche 454 GS Junior System. Image processing was performed by the GS RunBrowser software (Roche Applied Science). Methylation status of CpG sites was quantified using QUMA web‐based tool.

### 2.11 Statistical analysis

SPSS 22.0 software was used to statistical analyze all data. All data were expressed as mean ± SD of at least three separate experiments. For the RT‐PCR or Western blot analyses, the mean ± SD was derived from triplicate measurements of one experiment. A *t* test or one‐way ANOVA was used to compare the groups. *P* < .05 means significant difference.

## 3 RESULTS

### 3.1 Expression of GADD45γ gene in MDS patients' bone marrow cells and MDS cell lines

**FIGURE 1:**
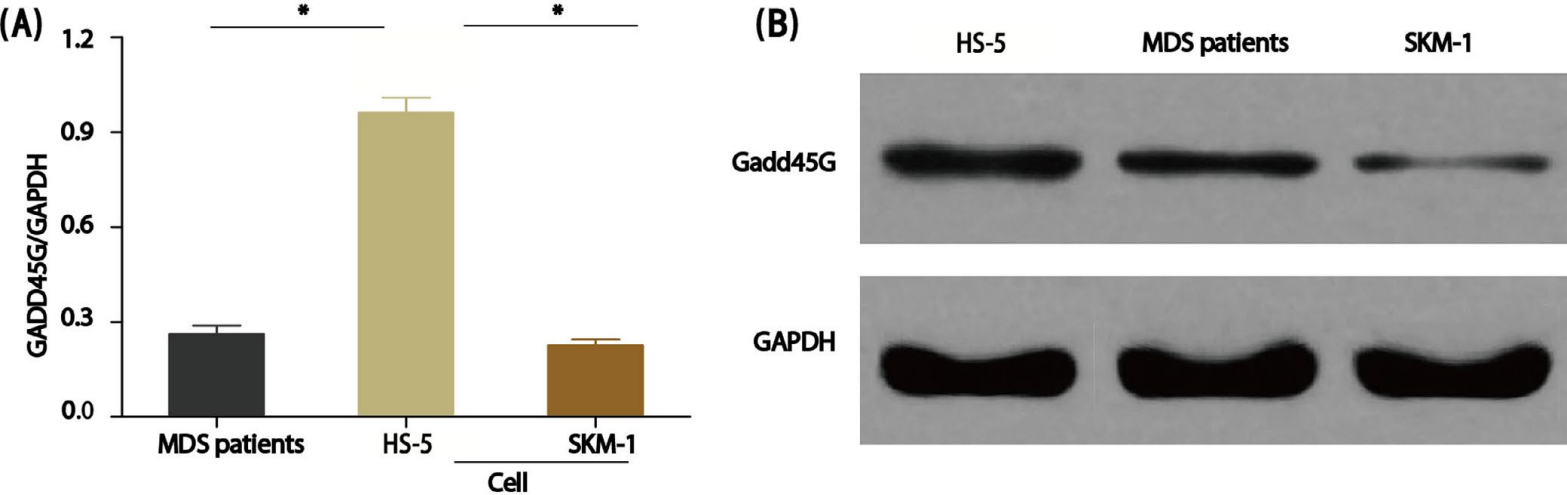
Expression comparison of GADD45γ in myelodysplastic syndrome (MDS) patients and MDS cell lines. (A) The mRNA expression of GADD45γ by RT‐PCR, *P < .01. (B) The protein expression of GADD45γ by Western blot

Based on the fact that GADD45γ could inhibit tumor growth by promoting apoptosis, and it had been reported that GADD45γ has biological activity in AML treatment, this study first analyzed the expression level of GADD45γ in MDS patients' bone marrow cells. Compared the expression level of GADD45γ in human acute myeloid leukemia cells SKM‐1 and human bone marrow stromal cell line HS‐5, it was found that the expression level of GADD45γ in SKM‐1 cell line had no significant difference between bone marrow cells in MDS patients, while the expression level of GADD45γ in HS‐5 cell line was significantly higher than the former two. The results showed that the expression level of GADD45γ was not high in MDS patients (Figure 1). According to the results of RT‐PCR and Western blot, GADD45γ protein may play a key role in the occurrence, development, and transformation of MDS.

### 3.2 Effection of GADD45γ on proliferation and apoptosis of MDS cells

**FIGURE 2:**
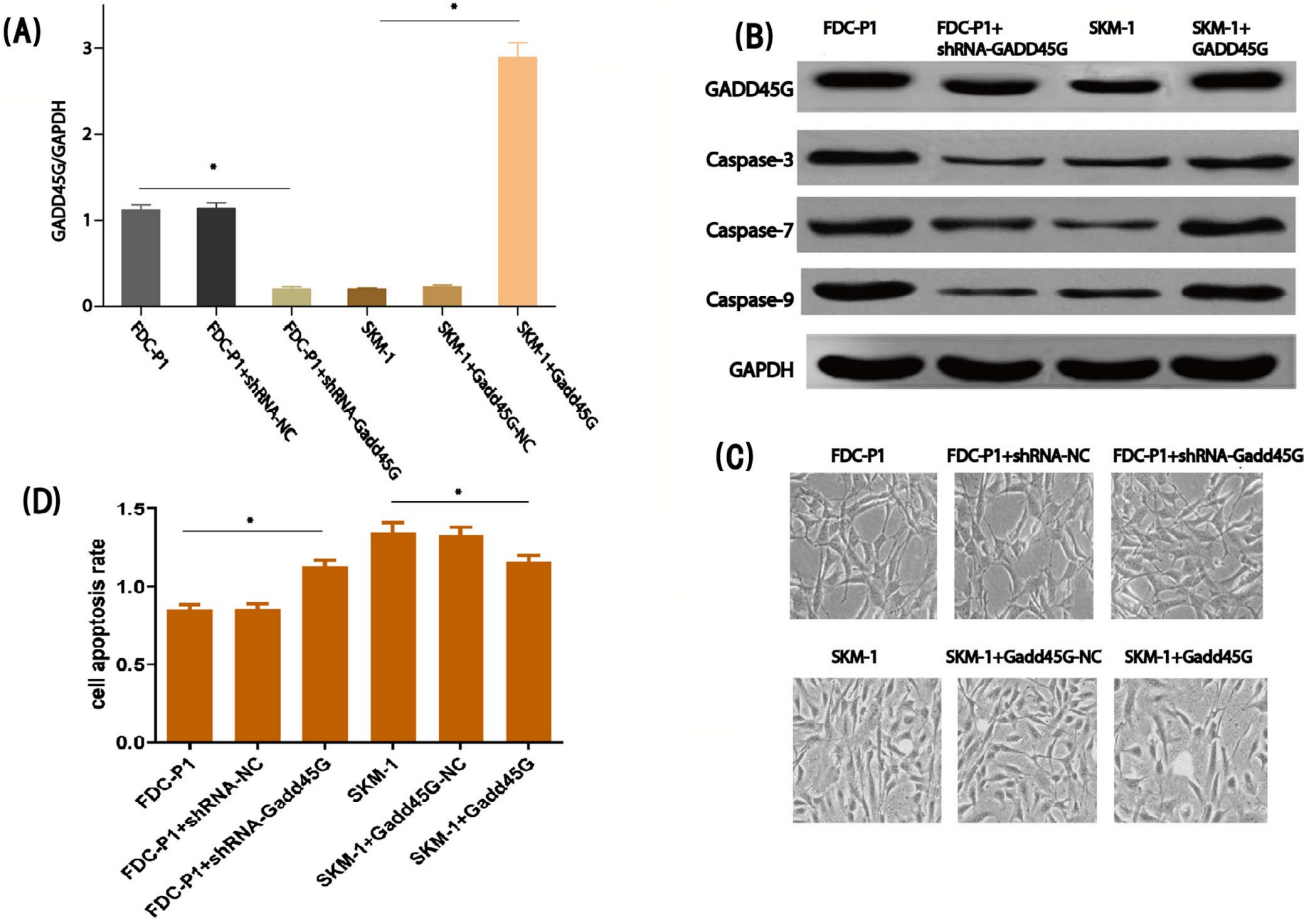
Effection of GADD45γ in myelodysplastic syndrome (MDS) cell lines. (A) The mRNA expression of GADD45γ in different cell lines by RT‐PCR, *P < .01. (B) The protein expression of GADD45γ and apoptosis‐related proteins in different cell lines by Western blot. (C) The cell proliferation rates of cells transfected with different plasmids,*P < .05. (D) The role of GADD45γ in apoptosis of SKM‐1 and FDC‐P1 cells

After that, we studied the biological activity of GADD45γ in MDS by transient expression of GADD45γ in SKM‐1 cells or inhibition of GADD45γ protein expression by shRNA in FDC‐P1 cells. The results showed that GADD45γ was successfully expressed in SKM‐1 cell line, and the expression level was significantly higher than that in the control group. The expression of GADD45γ was successfully inhibited in FDC‐P1 cells (Figure 2A and B). The expression level of apoptosis‐related protein, Caspase‐3, Caspase‐7, and Caspase‐9, was positive correlation with the expression level of GADD45γ in SKM‐1 cells and FDC‐P1 cells (Figure 2B). The proliferation rate of SKM‐1 cells is positive correlation and FDC‐P1 cells is negative correlation with the expression level of GADD45γ by CCK‐8 (Figure 2C). It indicated that decreasing GADD45γ expression reduced the proliferation of FDC‐P1 cells and raised the SKM‐1 cell proliferation. And meanwhile, the increasing GADD45γ expression raised the apoptosis of FDC‐P1 cells and lower the SKM‐1 cell apoptosis (Figure 2D). So far, we found that GADD45γ can inhibit or promote apoptosis by changing the expression level of apoptosis‐related proteins in MDS cell line.

### 3.3 Effection of azacitidine on the expression of GADD45γ in MDS cells

**FIGURE 3:**
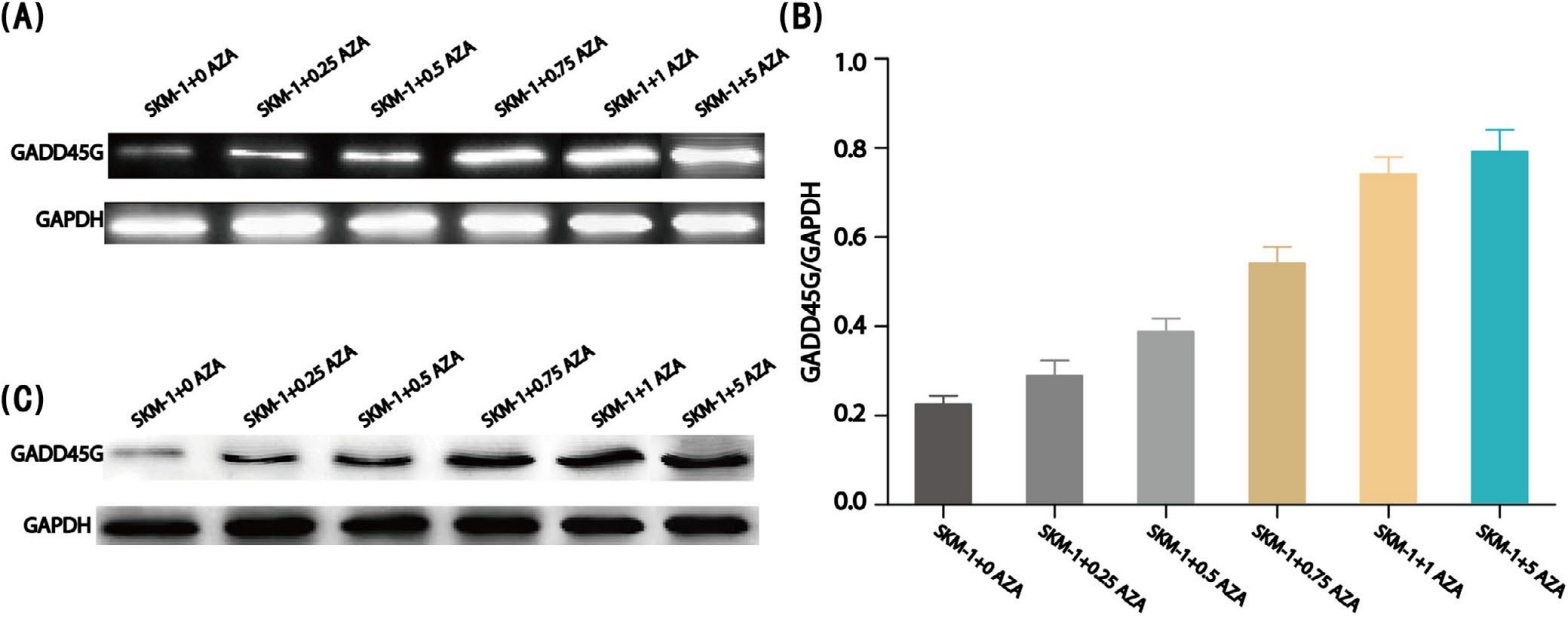
Effection of GADD45γ in myelodysplastic syndrome cell lines. (A) and (B) The mRNA expression of GADD45γ in SKM‐1 cells under different concentrations of AZA by RT‐PCR. (C) The protein expression of GADD45γ in SKM‐1 cells under different concentrations of AZA by Western blot. The final concentration of azacitidine in each experimental group was set as 0, 0.25, 0.5, 0.75, 1, and 5 μmol\L, respectively

Azacitidine was the first drug to treat MDS, but its mechanism needed further study. After confirming the effect of GADD45γ on MDS cell line by inducing apoptosis, we began to try to detect the change of GADD45γ protein expression level in MDS cell line affected by azacitidine. After treatment of SKM‐1 cell line with different concentration of azacitidine, we found that the proliferation of SKM‐1 cell line was significantly inhibited with the increase of azacitidine concentration, which was in direct proportion to the concentration of azacitidine. To detect the expression level of GADD45γ in SKM‐1 cell line after azacitidine treatment, we were surprised to find that with the increase of azacitidine concentration, the expression level of GADD45γ protein in the cell line also changed (Figure 3). When the concentration of azacitidine increased, the expression level of GADD45γ increased, the expression of apoptosis‐related proteins also increased, and the proliferation of MDS cell line was inhibited. So far, we had determined that azacitidine could promote the apoptosis of SKM‐1 cells by regulating the expression level of GADD45γ in MDS cells and changing the expression level of apoptosis‐related proteins.

### 3.4 Mechanism of azacitidine targeting GADD45γ gene therapy for MDS

**FIGURE 4:**
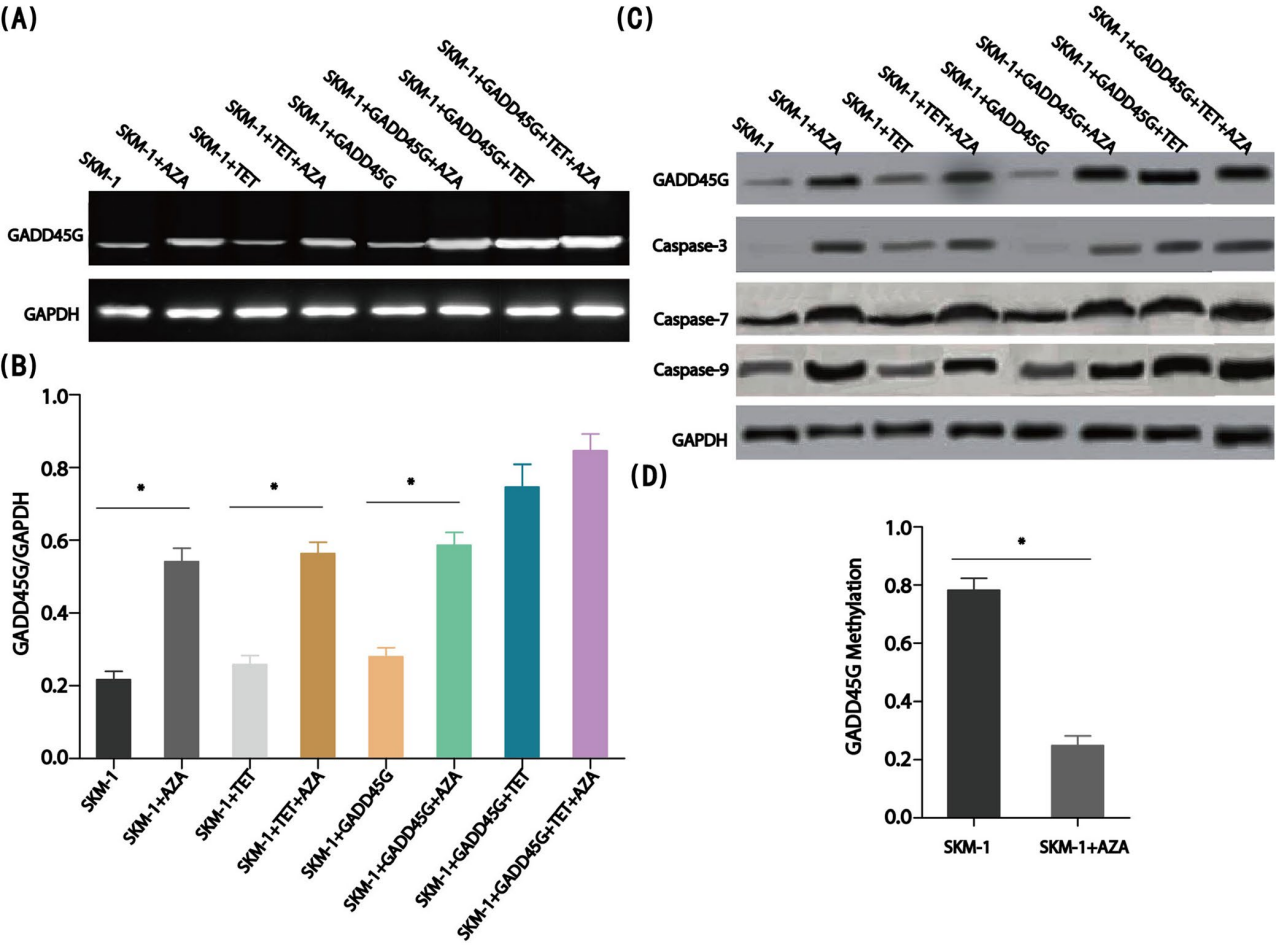
Effection of GADD45γ in myelodysplastic syndrome cell lines. (A) and (B) The mRNA expression of GADD45γ in SKM‐1 cells under AZA by RT‐PCR, *P < .01. (C) The protein expression of GADD45γ and apoptosis‐related proteins in SKM‐1 cells under AZA by Western blot. (D) Methylation of GADD45γ’s CpGs in the MDS cell line, *P < .01

In order to study how azacitidine acted on MDS cell line through GADD45γ, we further overexpressed GADD45γ in SKM‐1 cell line. The results showed that GADD45γ was overexpressed in SKM‐1 cell line, and with the increase of GADD45γ expression level, the protein expression level related to apoptosis was also increased, and the proliferation of SKM‐1 cell line was significantly inhibited (Figure 4). When azacitidine was added to SKM‐1 cell line, the expression level of GADD45γ was further increased, and the effect of apoptosis was more obvious.

Azacitidine is a classical demethylation drug. GADD45γ DNA was extracted from the experimental group and the control group. Compared with the control group, the methylation level of GADD45γ in the experimental group with azacitidine was significantly lower. So far, azacitidine can improve the expression level of GADD45γ by changing the methylation level of GADD45γ gene, promote cell apoptosis, promote MDS cell line apoptosis, and finally achieve the effect of MDS disease treatment. GADD45γ can be used as a target and biomarker for the treatment of MDS.

## 4 DISCUSSION

Through the analysis and comparison of MDS patients' bone marrow cell samples and SKM‐1 cell lines, we found that the expression level of GADD45γ protein in MDS patients was significantly lower than that of HS‐5. It was probably related to the occurrence, development, and transformation of MDS. If the bone marrow samples of healthy people could be obtained in the follow‐up study, compared with the expression level of GADD45γ in MDS patients, the role of GADD45γ in the occurrence and development of MDS could be further confirmed. In order to further analyze the role and influence of GADD45γ in the pathological process of MDS, the expression of GADD45γ in FDC‐P1 was silenced, resulting in accelerated cell proliferation and reduced apoptosis. The recombinant expression of GADD45γ in SKM‐1 cells showed that the increase of GADD45γ expression level could promote the apoptosis of SKM‐1 cells, inhibit the proliferation of SKM‐1 cells, and have certain value for the malignant transformation and prognosis of MDS. In later research, we could investigate whether GADD45γ could participate in autophagy of MDS‐related cell lines, and the application of GADD45γ in the treatment of MDS diseases.

In this study, we found that the expression of GADD45γ in MDS cell line was significantly increased when azacitidine was used as demethylation drug, and the dose of azacitidine was related to the apoptosis of MDS cell line and the expression of GADD45γ in MDS cell line. Furthermore, the overexpression of GADD45γ in SKM‐1 cell line was studied. The results showed that GADD45γ could induce the apoptosis of SKM‐1 cell line through caspase signaling pathway. When azacitidine was added, the expression level of GADD45γ in SKM‐1 cell line continued to increase, and the apoptosis was more obvious. Methylation analysis showed that the DNA methylation level of GADD45γ in SKM‐1 cell line was significantly higher than that in SKM‐1 control group with azacitidine added, while the expression level of GADD45γ in SKM‐1 cell line was significantly lower than that in SKM‐1 control group with azacitidine added, which was also confirmed in the GADD45γ overexpression system of SKM‐1 cell line, indicating that azacitidine can promote the expression of GADD45γ in MDS cell line, and the demethylation of GADD45γ could improve the expression level of GADD45γ. GADD45γ could further induce the apoptosis of MDS cell line through caspase signal pathway and finally achieve the effect of MDS treatment. Although azacitidine targeting GADD45γ had been proposed in this study to promote the apoptosis of MDS cell line through demethylation, more detailed apoptosis mechanism needs to be further studied, especially the specific cytokines involved in apoptosis signal pathway.

In summary, we found the apoptosis promoting effect of GADD45γ in MDS cell line and proposed the more detailed mechanism of azacitidine increasing the expression level of GADD45γ through demethylation to inhibit MDS cell proliferation and promote apoptosis. This provides a new perspective for MDS‐targeted therapy and proposes a new potential biomarker for MDS demethylation drug therapy. In addition, the overexpression of GADD45γ is expected to become a new method for MDS‐targeted therapy.

## CONFLICT OF INTEREST

The authors declare no competing financial interests.

## AUTHORS’ CONTRIBUTIONS

DL conceived and supervised the research. YY designed and performed the experiments, analyzed the data, and wrote the manuscript. JL, YG, and LL analyzed the data.
